# Supplementary material for: Key evolutionary events in the emergence of a globally disseminated, carbapenem resistant clone in the Escherichia coli ST410 lineage
Source: Commun Biol. 2019 Aug 29;2:322. doi: 10.1038/s42003-019-0569-1 (PMC6715731; doi:10.1038/s42003-019-0569-1)
Supplement: Supplementary file 1 — Supplementary Information [file 42003_2019_569_MOESM1_ESM.pdf]

Supplementary Figures

Supplementary Figure 1. Identification of recombination regions in ST410 *E. coli.* The figure is generated using Gubbins. The emerging clade identified in this study is depicted in cyan. The complete chromosome sequence of strain 020026 was used as the reference. Strains of the B4/H24RxC MDR clone are shown in cyan. A 13-kb region unique to the B4/H24RxC MDR clone is indicated by an arrow.

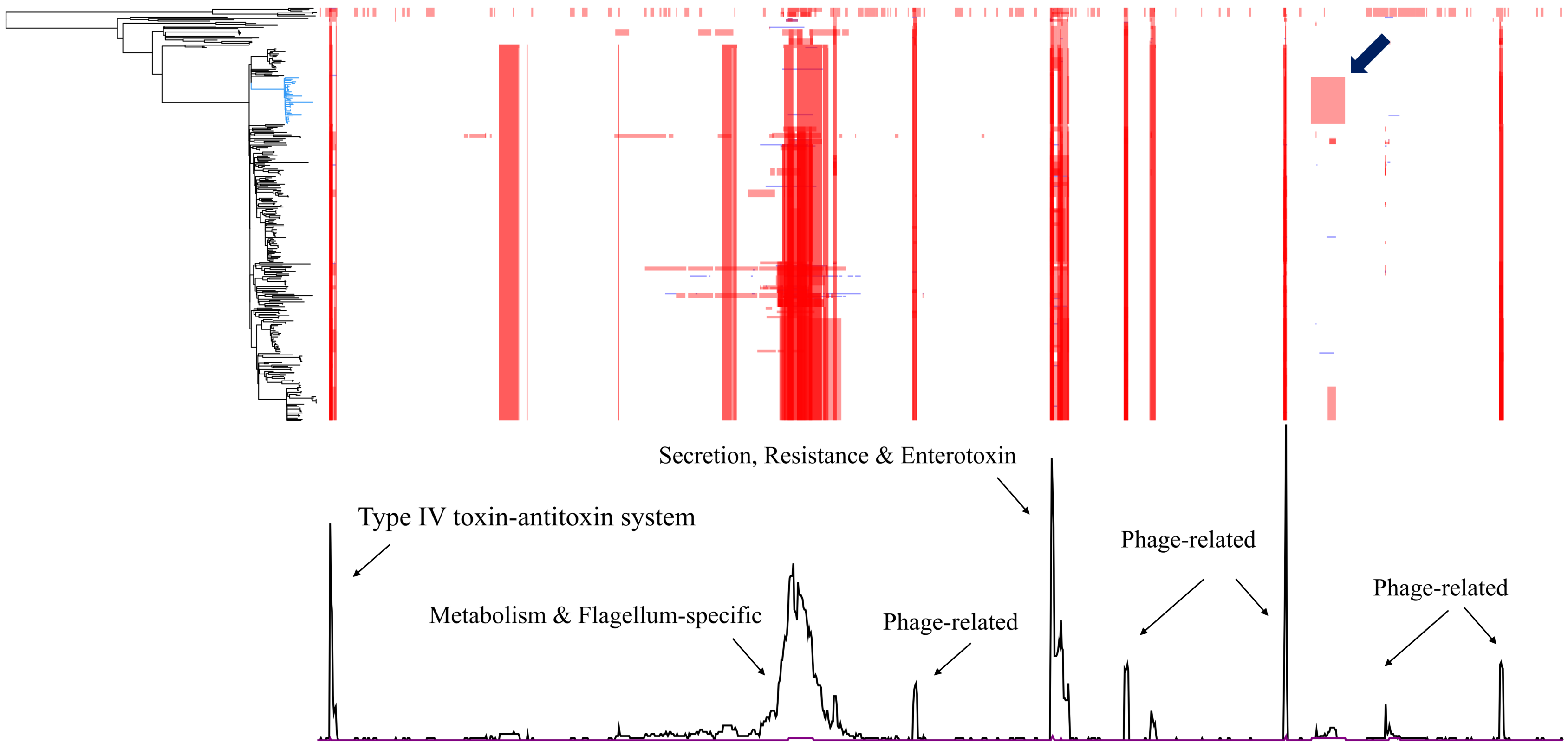

Supplementary Figure 2. The re-constructed phylogenomic tree of the B4/H24RxC MDR clone, its closely-related strains and the sister clade. The phylogenomic tree is rooted on a strain from the sister clade. Panel A, the original phylogenomic tree with bootstraps being represented by gradient colours on the branches. Panel B, the same tree with transformed topology by ignoring branch lengths for a clearer illustration. Bootstraps are presented by numbers on the branches. For both panel A and B, the pink region indicates strains within the B4/H24RxC clone (n=37), the yellow region indicates those closely-related ones (n=6) and the green region indicates the one (accession no. ERR1197948) from the sister clade, which was used for rooting.

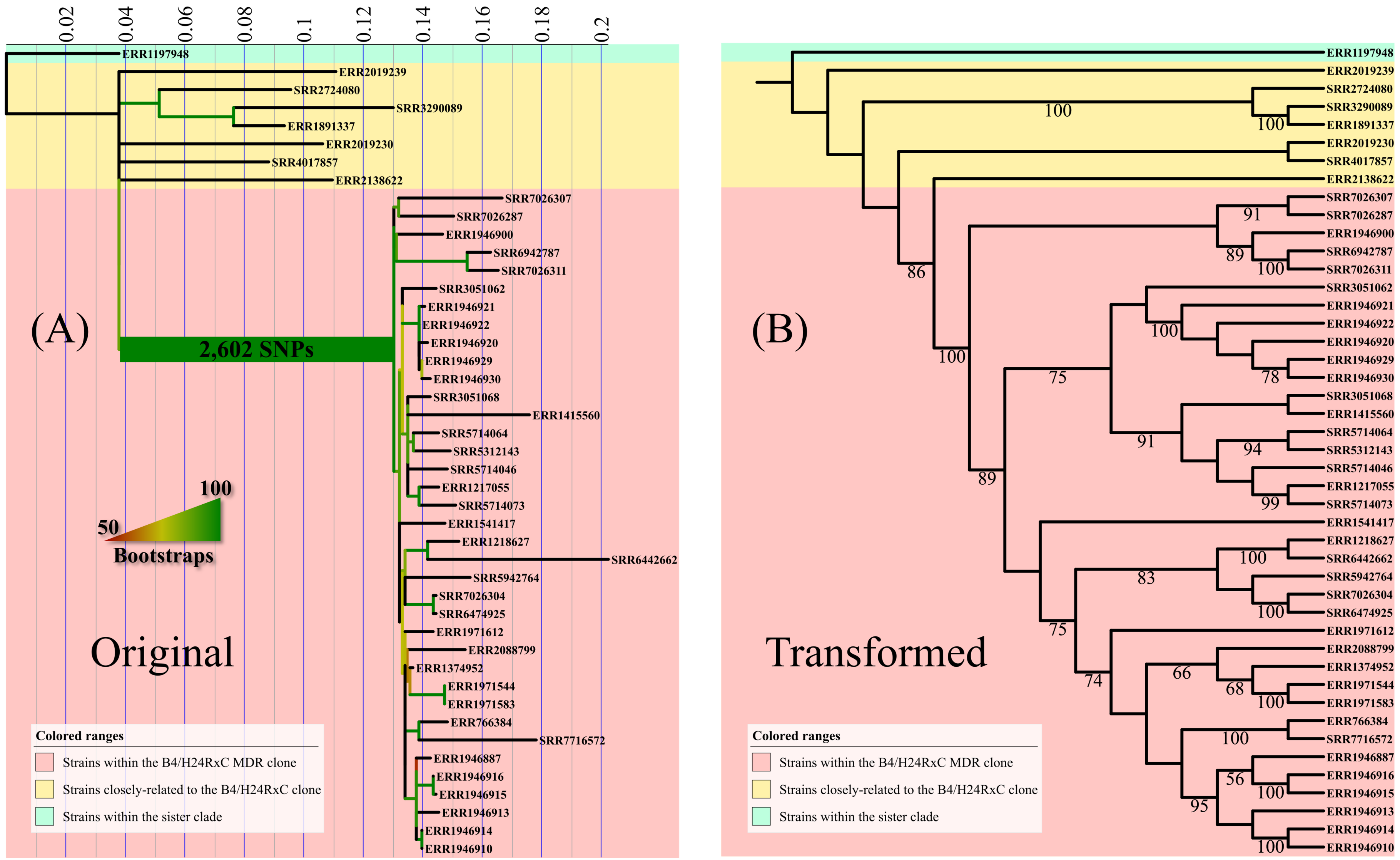

(A)
Original
2,602 SNPs
100
50
Bootstraps
Colored ranges
Strains within the B4/H24RxC MDR clone
Strains closely-related to the B4/H24RxC clone
Strains within the sister clade
(B)
Transformed

Supplementary Figure 3. Coalescent analysis with all dated strains failed to converge within an applicable time. This dated phylogenomic tree of ST410 *E. coli* strains was constructed using BactDating v1.0.1 and corrected for recombination using Gubbins v2.3.4. The run was under mixed model with 10^8 iterations to ensure that the Markov chain Monte Carlo (MCMC) was run for long enough to converge (the effective sample size of the inferred parameters α, μ and σ were > 200). However, as shown in the figure, α and μ were < 200, suggesting that the coalescent analysis with all dated strains had not been run for long enough, indicating the estimated posteriors were less reliable. Therefore, another coalescent analysis (Figure 3 in the article) was performed by excluding four distant strains within the B4/H24RxC MDR clone. The four strains are strain KOEGE 131 (358a) (accession no. SRR785629), MOD1-EC5419 (accession no. SRR6512532), KTE221 (accession no. SRR633754) and NC_STEC121 (accession no. SRR5470036) and have distant relationship to the remaining clonal strains (>5,000 SNPs with almost all other ST410 strains).

alpha = 3.19e+02 [2.46e+02;4.03e+02] ESS = 189.0926
mu = 3.99e+00 [3.27e+00;4.70e+00] ESS = 129.9924
sigma = 1.61e+00 [1.20e+00;2.09e+00] ESS = 204.7422

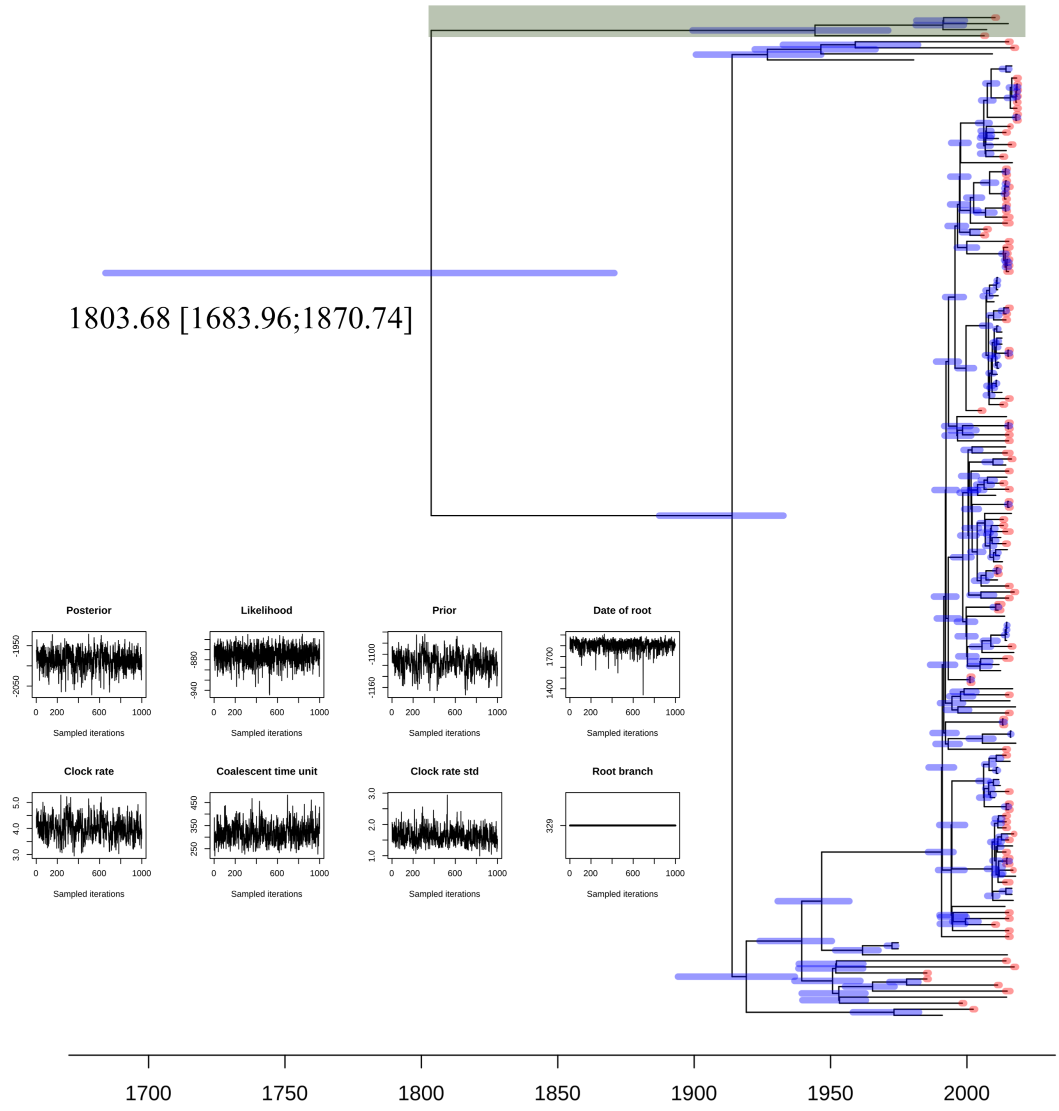

Supplementary Figure 4. Alignment of *fhuA* gene sequence between strain 020026 of the emerging clone and strain 020001, a representative of other clones within ST410.

```
020001 FhuA    1 MARSKTAQPKHSLRKIAVVVATAVSGMSVYAQAAVEPKEDTITVTAAPAPQESAWGPAAT
020026 FhuA    1 .................................................S..........

020001 FhuA   61 IAAKHSATATKTDTPIEKTPQSISVVTNEEMQMHQFQSVKEALGYTPGVTVSSRGASNTY
020026 FhuA   61 ...RQ...G.......Q.V........A...AL..PK......S.....S.GT.......

020001 FhuA  121 DFVIIRGFSSVGLNQNNYLDGLKLQGNFYNDAVIDPYMLERVELMRGPTSVLYGKSNPGG
020026 FhuA  121 .HL.....AAE.QS.....N.....................A.I....V.......S...

020001 FhuA  181 IISMVSKRPTTEPLKEIQFKMGTDNLFQTGFDFSDALDDNGEFSYRLTGLARSTNEQQKN
020026 FhuA  181 LLN.............V...A...S..........S...D.VY..........A.A...G

020001 FhuA  241 SESQRYTIAPSFSWRPDDKTNFTFLSYFQNEPETGYYGWLPKEGTVEPLPNGKRLPTDFN
020026 FhuA  241 ..E...A...A.T...............................................

020001 FhuA  301 EGASNNTYSRNQKMVGYSFEHGFNDTFTVRQNLRFSEMKTSQKSVYGTGIAND-------
020026 FhuA  301 ...K.......E.......D.E.............A.N....N....Y.VCS.PANAYSK

020001 FhuA  354 ----------GHTLNRGTVVDNERLQNFSVDTQLESKFATGEVEHTLLTGVDFMRMRNDI
020026 FhuA  361 QCAALAPADK..Y.A.KY...D.K..........Q......DID................

020001 FhuA  404 NASFGSAPSIDLYNKYHPEYFAF-------GNAEPYQ-MNESKQTGIYVQDQAEWNKWVF
020026 FhuA  421 ..W..YDD.VP.L.L.N.VNTD.DFNAKDPA.SG..RIL.KQ....V......Q.D.VLV

020001 FhuA  456 TLGGRYDWSKQATTVRENSYTPTEGYIERNDHQFTWRGGVNYLFDNGISPYFSYSQSFEP
020026 FhuA  481 ........AD.ESLN.VAGT.D-----K.D.K...............VT......E....

020001 FhuA  516 SAFDLWSNPRVS-----YKPSKGEQYEAGVKYVPNDMPVVVTGAVYQLTKTNNLTADPTN
020026 FhuA  536 .S-------Q.GKDGNIFA....K...V......E.R.I.......N.......M...EG

020001 FhuA  571 PLAQVPAGEIRARGVELEAKAALNANINLTASYTYTDAEYTKDTNLKGKTPEQVPEHMAS
020026 FhuA  589 SFFS.EG.........I......S.SV.VVG..........T..TY..N..A...K....

020001 FhuA  631 LWGDYTFNEGPLSGLTLGTGGRFIGSSYGDPANTFKVGSAAVMDAVVKYDLARFGMAGSS
020026 FhuA  649 ..A....FD.............YT.........S.....YT.V..L.R.....V.....N

020001 FhuA  691 LAVNVNNLLDREYVASCFQTYGCFWGAERQVVATATFRF
020026 FhuA  709 V.LH....F.........N....................
```

Supplementary Tables

Supplementary Table 1. Basic information of the eight hospitals in this study.

Hospital	Bed capacity	Hospital	Place
West China Hospital, Sichuan University	4,300	Referal	Chengdu City
The Sixth People’s Hospital of Chengdu City	800	Municipal	Chengdu City
Meishan Hospital of Traditional Chinese Medicine	1,610	Municipal	Meishan City
Mianyang Central Hospital	1,500	Municipal	Mianyang City
The First People’s Hospital of Liangshan Yi Autonomous Prefecture	1,389	Municipal	Xichang City, Liangshan Yi Autonomous Prefecture
The Second People’s Hospital of Yibin City	2,100	Municipal	Yibin City
The First People’s Hospital of Zigong City	1,800	Municipal	Zigong City
The People's Hospital of Leshan City	1,620	Municipal	Leshan City

All hospitals are general hospitals in Sichuan province, China.

Supplementary Table 2. SNPs between ST167 strains in this study.

	020007	020016	020033	020076
020007	-	558	174	707
020016	558	-	603	425
020033	174	603	-	716
020076	707	425	716	-

Supplementary Table 3. SNPs between ST617 strains in this study.

	020023	020044	020085	020141	020149
020023	-	409	409	409	524
020044	409	-	0	0	676
020085	409	0	-	0	676
020141	409	0	0	-	676
020149	524	676	676	676	-

Supplementary Table 4. The yield of MinION reads of strain 020001, strain 020026 and strain 020032.

Strain	020001	020026	020032
Mean read length (bp)	6,633.3	8,124.1	3,752.7
Mean read quality (Q)	10	9.9	9.9
Number of reads	25,399	119,559	226,388
Read length N*50* (bp)	8,288	9,710	5,104
Total bases (bp)	168,479,767	971,305,509	849,565,943
> Q7	100%	100%	100%
> Q10	51.4%	49.4%	52.2%

Supplementary Table 5. The complete genome and antimicrobial resistance genes of strain 020001, strain 020026 and strain 020032.

	Accession no.	Size, bp	Replicon type	Genes mediating resistance to						
				β-lactam	Aminoglycoside	Macrolide	Rifampin	Sulfonamide	Tetracycline	Trimethoprim
020001										
Chromosome	CP032426	4,908,742	-							
p1_020001	CP032420	2,959	ColRNAI							
p2_020001	CP032421	2,444	ND							
p3_020001	CP032422	1,989	ColpVC							
p4_020001	CP032423	1,521	ND							
pOXA1_020001	CP032424	88,392	IncFIA, IncFIB, IncFIC	$bla_{\text{OXA-1}}$	*aac(6')-Ib-cr*		*catB3*		*tet*(A)	
pNDM5_020001	CP032425	46,161	IncX3	$bla_{\text{NDM-5}}$						
020026										
Chromosome	CP034958	4,763,166	-	$bla_{\text{CMY-2}}$						
p1_020026	CP034954	4,097	ND							
p2_020026	CP034955	2,088	Col(BS512)							
pCTXM15_020026	CP034956	98,397	IncFIA, IncFII, IncQ1	$bla_{\text{CTX-M-15}}$, $bla_{\text{OXA-1}}$, $bla_{\text{TEM-1}}$	*aac(3)-IId*, *aadA5*, *aph(3'')-Ib*, *aph(6)-Id*	*mph*(A)		*sul1*, *sul2*	*tet*(B)	*dfrA17*
pNDM5_020026	CP034957	46,161	IncX3	$bla_{\text{NDM-5}}$						
020032										
Chromosome	CP034966	4,686,461	-							
p1_020032	CP034959	90,842	ND							
p2_020032	CP034960	5,167	Col156							
p3_020032	CP034961	2,101	Col(BS512)							
p4_020032	CP034962	2,058	ColpVC							
pCMY42_020032	CP034963	38,448	IncI1	$bla_{\text{CMY-42}}$						
pCTXM3_020032	CP034964	87,704	IncFII	$bla_{\text{CTX-M-3}}$	*aac(3)-IId*, *aadA5*	*erm(B)*, *mph*(A)		*sul1*		*dfrA17*
pNDM5_020032	CP034965	46,161	IncX3	$bla_{\text{NDM-5}}$						

ND, undetermined.

Supplementary Table 6. Phage regions of strain 020001 predicted by PHASTER.

Region	Region Length	Completeness	Score	No. of Proteins	Region Position	Most Common Phage	GC %
1	5.5Kb	incomplete	30	9	195210-200735	PHAGE_Entero_933W_NC_000924(2)	47.5
2	15.4Kb	incomplete	50	12	913815-929273	PHAGE_Cronob_vB_CsaM_GAP32_NC_019401(1)	45
3	39Kb	intact	120	26	1954948-1993973	PHAGE_Entero_Sf101_NC_027398(9)	48.8
4	27.3Kb	incomplete	30	22	2463721-2491079	PHAGE_Entero_mEp460_NC_019716(4)	46.7
5	29.3Kb	questionable	70	40	3307859-3337215	PHAGE_Entero_BP_4795_NC_004813(10)	45.1
6	36.4Kb	intact	100	35	3818437-3854872	PHAGE_Entero_mEp460_NC_019716(16)	49.4

Supplementary Table 7. Stability of pNDM5_020026 and pOXA181 in *E. coli* J53

Plasmid and medium	Biological replicate[1]	Technical replicate	Stability frequency	Mean± SD
pNDM5-020026 LB Medium	1	1	0.980	0.980±0.004
		2	0.977	
		3	0.984	
	2	1	1.010	0.995±0.013
		2	0.987	
		3	0.988	
	3	1	0.983	0.995±0.012
		2	1.006	
		3	0.998	
pNDM5-020026 M9 Medium	1	1	0.883	0.903±0.017
		2	0.912	
		3	0.914	
	2	1	0.866	0.881±0.013
		2	0.89	
		3	0.886	
	3	1	0.884	0.877±0.013
		2	0.862	
		3	0.884	
pOXA181 LB Medium	1	1	0.985	0.996±0.010
		2	1.005	
		3	0.998	
	2	1	0.993	0.993±0.001
		2	0.993	
		3	0.994	
	3	1	0.995	1.015±0.022
		2	1.038	
		3	1.013	
pOXA181 M9 Medium	1	1	0.898	0.925±0.026
		2	0.927	
		3	0.949	
	2	1	0.908	0.921±0.014
		2	0.936	
		3	0.92	
	3	1	0.922	0.924±0.005
		2	0.92	
		3	0.93	

[1]The difference of stability frequency among biological replicates was not statistically significant (P>0.05).

Supplementary Table 8. Biofilm formation assay results

Strain	$OD_{590\ nm}$ (mean±SD)	F	Df	P[1]	P[2]	$OD_{595\ nm}$ (mean±SD)	F	Df	P[1]	P[2]
**020026**	**0.299±0.045**	**24.48**	**2**	**<0.001**	**-**	**0.290±0.034**	**31.00**	**2**	**<0.001**	**-**
**020001**	**0.408±0.105**				**<0.001**	**0.393±0.091**				**<0.001**
020032	0.276±0.059				0.265	0.258±0.059				0.081
ATCC 19606	0.393±0.029	-	-	-	-	0.373±0.025	-	-	-	-
H_2O	0.047±0.004	-	-	-	-	0.045±0.004	-	-	-	-

[1]The differences of absorption values at both $OD_{590\ nm}$ and $OD_{595\ nm}$ among three strains (020001, 020026, 020032) were compared with one-way ANOVA. Those with statistical significance (*P*<0.05) are highlighted in bold.

[2]The differences of absorption values at both $OD_{590\ nm}$ and $OD_{595\ nm}$ between strain 020026 and 020001 or between strain 020026 and 020032 were compared with LSD (Least-significant Difference). Those with statistical significance (*P*<0.05) are highlighted in bold.

Supplementary Table 9. Survival (number of larvae) of *G. mellonella* at 72 h after infection by bacterial strains.

Strain	Inoculums (CFU)			
	1×10^4	1×10^5	1×10^6	1×10^7
020026	75.0%	48.8%	11.3%	0.0%
020001	83.8%	30.0%	16.3%	0.0%
020032	67.5%	42.5%	26.3%	7.5%
KP767	58.8%	43.8%	2.5%	0.0%
DH5α	100.0%	100.0%	100.0%	100.0%

Supplementary Table 10. Relative fitness of strain 020026 compared to strain 020001 and strain 020032

Strain, with/without DIP	Biological Replicate[1]	Technical replicate	Relative fitness (w)	Mean± SD
020026vs020001 without DIP	1	1	0.945	0.945±0.008
		2	0.937	
		3	0.953	
	2	1	0.947	0.933±0.019
		2	0.911	
		3	0.941	
	3	1	0.903	0.923±0.211
		2	0.92	
		3	0.945	
020026vs020001 with DIP	1	1	1.08	1.070±0.009
		2	1.065	
		3	1.064	
	2	1	1.097	1.071±0.022
		2	1.058	
		3	1.059	
	3	1	1.048	1.068±0.020
		2	1.069	
		3	1.087	
020026vs020001 without DIP	1	1	1.595	1.578±0.049
		2	1.523	
		3	1.616	
	2	1	1.517	1.539±0.043
		2	1.589	
		3	1.511	
	3	1	1.53	1.540±0.028
		2	1.571	
		3	1.518	

[1]The difference of w values among biological replicates was not statistically significant (P>0.05).
