# Supplementary material for: Key evolutionary events in the emergence of a globally disseminated, carbapenem resistant clone in the Escherichia coli ST410 lineage
Source: Commun Biol. 2019 Aug 29;2:322. doi: 10.1038/s42003-019-0569-1 (PMC6715731; doi:10.1038/s42003-019-0569-1)
Supplement: Supplementary file 2 — Description of additional supplementary items [file 42003_2019_569_MOESM2_ESM.pdf]

Description of additional supplementary items

Supplementary Dataset 1. CREC strains in this study.
Supplementary Dataset 2. ST410 *E. coli* genomes available in NCBI SRA database (accessed by August 1, 2018).
Supplementary Dataset 3. SNPs between ST410 genomes using strain 020001 as the reference.
Supplementary Dataset 4. SNPs between ST410 genomes using strain 020026 as the reference.
Supplementary Dataset 5. SNPs unique for this emerging clone.
