# Supplementary material for: Key evolutionary events in the emergence of a globally disseminated, carbapenem resistant clone in the Escherichia coli ST410 lineage
Source: Commun Biol. 2019 Aug 29;2:322. doi: 10.1038/s42003-019-0569-1 (PMC6715731; doi:10.1038/s42003-019-0569-1)
Supplement: Supplementary file 8 — Reporting Summary [file 42003_2019_569_MOESM8_ESM.pdf]

nature research

Corresponding author(s): Zhiyong Zong

Last updated by author(s): Jul 22, 2019

Reporting Summary

Nature Research wishes to improve the reproducibility of the work that we publish. This form provides structure for consistency and transparency in reporting. For further information on Nature Research policies, see Authors & Referees and the Editorial Policy Checklist.

Statistics

For all statistical analyses, confirm that the following items are present in the figure legend, table legend, main text, or Methods section.

n/a	Confirmed	
☐	☒	The exact sample size (n) for each experimental group/condition, given as a discrete number and unit of measurement
☐	☒	A statement on whether measurements were taken from distinct samples or whether the same sample was measured repeatedly
☐	☒	The statistical test(s) used AND whether they are one- or two-sided<br>*Only common tests should be described solely by name; describe more complex techniques in the Methods section.*
☐	☒	A description of all covariates tested
☒	☐	A description of any assumptions or corrections, such as tests of normality and adjustment for multiple comparisons
☐	☒	A full description of the statistical parameters including central tendency (e.g. means) or other basic estimates (e.g. regression coefficient) AND variation (e.g. standard deviation) or associated estimates of uncertainty (e.g. confidence intervals)
☐	☒	For null hypothesis testing, the test statistic (e.g. F, t, r) with confidence intervals, effect sizes, degrees of freedom and P value noted<br>*Give P values as exact values whenever suitable.*
☒	☐	For Bayesian analysis, information on the choice of priors and Markov chain Monte Carlo settings
☒	☐	For hierarchical and complex designs, identification of the appropriate level for tests and full reporting of outcomes
☒	☐	Estimates of effect sizes (e.g. Cohen's d, Pearson's r), indicating how they were calculated

Our web collection on statistics for biologists contains articles on many of the points above.

Software and code

Policy information about availability of computer code

Data collection	NA
Data analysis	SPAdes v3.13.0, Unicycler v0.4.7, Plion v1.22, Prokka v1.13, BactDating v1.0.1, BWA v0.7.17, the PHASTER webserver, Cutadapt v1.16, BBTools v37.92, RAxML v8.2.12, iTOL v3, Phandango v1.3.0, Roary v3.12.0, Scoary v1.6.16, PlasmidFinder 2.0 , ResFinder 3.1, Guppy v3.0.3, Porechop v0.2.4, Snippy v4.3.6, Gubbins v2.3.4, snp-dists v0.6.3, FimTyper 1.0, the BPROM webserver,Bandage v0.8.1, BLAST v2.7.1, SPSS v21.0

For manuscripts utilizing custom algorithms or software that are central to the research but not yet described in published literature, software must be made available to editors/reviewers. We strongly encourage code deposition in a community repository (e.g. GitHub). See the Nature Research guidelines for submitting code & software for further information.

Data

Policy information about availability of data

All manuscripts must include a data availability statement. This statement should provide the following information, where applicable:

- Accession codes, unique identifiers, or web links for publicly available datasets
- A list of figures that have associated raw data
- A description of any restrictions on data availability

All data generated or analyzed during this study are included in this article and its supplementary files.
Figures 1, 2, 3 and 5 are associated with raw data, which are available as Supplementary datasets. The raw results of Figure 4 are shown as Supplementary tables.
Nucleotide accession no. Draft genome sequences and short reads of the strains have been deposited in GenBank with the accession numbers being listed in Table 1. The complete sequences of the chromosome and plasmids of strain 020001 have been deposited in GenBank with the accession numbers CP032420 to CP032426.

Field-specific reporting

Please select the one below that is the best fit for your research. If you are not sure, read the appropriate sections before making your selection.

☒ Life sciences ☐ Behavioural & social sciences ☐ Ecological, evolutionary & environmental sciences

For a reference copy of the document with all sections, see nature.com/documents/nr-reporting-summary-flat.pdf

Life sciences study design

All studies must disclose on these points even when the disclosure is negative.

Sample size	25 strains in this study and 327 bacterial genomes retrieved from GenBank.
Data exclusions	NA
Replication	For biofilm formation assays, all experiments were performed in triplicate (biological replicates) and for each replicate, experiments were repeated nine times (technical replicates). For plasmid stability testing, the experiments for both pNDM5_020026 and pOXA-181 in LB broth and M9 media were all performed in triplicate (biological replicates). All experiments of virulence assay using wax moth were performed with five biological replicates, while iron source growth assay was performed in triplicate (biological replicates). For head to head competition, all experiments were performed in triplicate (biological replicates) and each biological replicate was repeated three times (technical replicates).
Randomization	NA
Blinding	NA

Reporting for specific materials, systems and methods

We require information from authors about some types of materials, experimental systems and methods used in many studies. Here, indicate whether each material, system or method listed is relevant to your study. If you are not sure if a list item applies to your research, read the appropriate section before selecting a response.

Materials & experimental systems

n/a	Involved in the study	
☒	☐	Antibodies
☒	☐	Eukaryotic cell lines
☒	☐	Palaeontology
☐	☒	Animals and other organisms
☒	☐	Human research participants
☒	☐	Clinical data

Methods

n/a	Involved in the study	
☒	☐	ChIP-seq
☒	☐	Flow cytometry
☒	☐	MRI-based neuroimaging

Animals and other organisms

Policy information about studies involving animals; ARRIVE guidelines recommended for reporting animal research

Laboratory animals	The study did not involve laboratory animals.
Wild animals	The study did not involve wild animals.
Field-collected samples	The study did not involve field-collected samples.
Ethics oversight	The ethical committee of West China Hospital.

Note that full information on the approval of the study protocol must also be provided in the manuscript.
